# Supplementary material for: SyNDI: synchronous network data integration framework
Source: BMC Bioinformatics. 2018 Nov 6;19:403. doi: 10.1186/s12859-018-2426-5 (PMC6219086; doi:10.1186/s12859-018-2426-5)
Supplement: Supplementary file 5 — Files of the example presented in Additional file 4. This additional file is a zip package that contains all files used in Additional file 4. Detailed descriptions of each of these files are presented in a README file included in this zip package. (GZ 30411 kb) [file 12859_2018_2426_MOESM5_ESM.gz › SyNDITools/Meme2Fimo/meme_4.9.0_4.tar.gz!/meme_4.9.0/website/html/motif_search.html]

MEME Suite - Submit A Job


|  |  |
| --- | --- |
|  | MEME Suite The MEME Suite web application requires the use of JavaScript   Javascript doesn't seem to be available on your browser.  Documentation on Motif Search Tools  The MEME Suite allows you to search databases of protein sequences in different ways using motifs as queries. Click on each of the tools listed below for more information on the different kinds of searches you can do.   - FIMO   finds matches to **individual motifs** - MAST   finds sequences that match a **set of motifs** - GLAM2SCAN   finds matches to a single **gapped motif** |
